# DNA Methylation and Expression Profiles of Whole Blood in Parkinson’s Disease

**DOI:** 10.3389/fgene.2021.640266

**Published:** 2021-04-26

**Authors:** Adrienne R. Henderson, Qi Wang, Bessie Meechoovet, Ashley L. Siniard, Marcus Naymik, Matthew De Both, Matthew J. Huentelman, Richard J. Caselli, Erika Driver-Dunckley, Travis Dunckley

**Affiliations:** ^1^Neurodegenerative Disease Research Center, Biodesign Institute, Arizona State University, Tempe, AZ, United States; ^2^Neurogenomics Division, Translational Genomics Research Institute, Phoenix, AZ, United States; ^3^Division of Neurology, Mayo Clinic, Scottsdale, AZ, United States

**Keywords:** Parkinson’s, DNA methylation, RNA-seq, biomarker, Parkinson’s and related diseases, mRNA-seq, expression profiling, methylation profiling

## Abstract

Parkinson’s disease (PD) is the second most common age-related neurodegenerative disease. It is presently only accurately diagnosed at an advanced stage by a series of motor deficits, which are predated by a litany of non-motor symptoms manifesting over years or decades. Aberrant epigenetic modifications exist across a range of diseases and are non-invasively detectable in blood as potential markers of disease. We performed comparative analyses of the methylome and transcriptome in blood from PD patients and matched controls. Our aim was to characterize DNA methylation and gene expression patterns in whole blood from PD patients as a foundational step toward the future goal of identifying molecular markers that could predict, accurately diagnose, or track the progression of PD. We found that differentially expressed genes (DEGs) were involved in the processes of transcription and mitochondrial function and that PD methylation profiles were readily distinguishable from healthy controls, even in whole-blood DNA samples. Differentially methylated regions (DMRs) were functionally varied, including near transcription factor nuclear transcription factor Y subunit alpha (*NFYA*), receptor tyrosine kinase *DDR1*, RING finger ubiquitin ligase (*RNF5*), acetyltransferase *AGPAT1*, and vault RNA *VTRNA2-1*. Expression quantitative trait methylation sites were found at long non-coding RNA *PAX8-AS1* and transcription regulator *ZFP57* among others. Functional epigenetic modules were highlighted by *IL18R1*, *PTPRC*, and *ITGB2*. We identified patterns of altered disease-specific DNA methylation and associated gene expression in whole blood. Our combined analyses extended what we learned from the DEG or DMR results alone. These studies provide a foundation to support the characterization of larger sample cohorts, with the goal of building a thorough, accurate, and non-invasive molecular PD biomarker.

## Introduction

Parkinson’s disease (PD) is a common neurodegenerative disease, second in prevalence only to Alzheimer’s disease (Mayeux and Stern, 2012; Tysnes and Storstein, 2017). It is projected to afflict nearly one million people in the US alone by 2020. PD characteristically manifests as overt motor defects following the destruction of dopaminergic neurons in the substantia nigra and is pathologically associated with α-synuclein protein aggregation into intracellular cytoplasmic inclusions, termed Lewy bodies. This brain pathology may result from initial insults *via* olfaction or in the gut, with retrograde trafficking into the affected brain regions (Hawkes et al., 2009; Planken et al., 2017). Neurodegeneration occurs early in the dopaminergic neurons of the substantia nigra, but Lewy body pathology occurs in limbic and cortical areas as PD progresses (Mattila et al., 2000).

Currently, PD diagnosis is predominantly based on the clinical manifestations of the disease, namely, by the findings of tremor, rigidity, and bradykinesia. Extrastriatal, non-motor symptoms of PD, including cognitive problems, apathy, depression, anxiety, hallucinations, and psychosis as well as sleep disorders, fatigue, autonomic dysfunction, sensory problems, and pain, can begin years before diagnosis, accompany the course of disease progression, and are major factors in reduced quality of life (Barone et al., 2009). The clinical diagnostic accuracy of 53% in these early stages of PD is unacceptably low (Mehta and Adler, 2016). Ongoing prevention therapy research is currently underway (Olanow and Schapira, 2013) and would be greatly facilitated by increased diagnostic accuracy at early-stage PD. Indeed, currently on average, over half of all dopaminergic neurons in the substantia nigra are already lost by the time of accurate clinical diagnosis (Delenclos et al., 2016), making prevention approaches problematic. A combination of multiple biomarker approaches as a diagnostic panel could accelerate improvements in early diagnostic accuracy. This will be important in pushing the point at which diagnosis or high-risk prediction can be made to an even earlier time point in pre-motor prodromal stages. To this end, multiple pre-motor biomarkers are actively being investigated for their potential to identify early-stage PD or patients at risk for developing PD (Haas et al., 2012), including clinical measures (rapid eye movement behavior disorder, olfactory deficits, mood disorders), molecular measures (α-syn in cerebrospinal fluid and blood), and brain imaging.

Epigenetic factors, which can be modified by both environment and ongoing disease processes, are emerging as important components of neurodegenerative diseases, including PD (Pavlou and Outeiro, 2017). The dynamic process of DNA methylation is one of the most commonly studied epigenetic regulators in human disease. Addition of a methyl group primarily occurs on cytosine bases that are next to guanines, referred to as CpG sites, but methylation can also occur to a much lesser extent on cytosines next to other bases (Messerschmidt et al., 2014). Initial hypotheses regarding methylation function were mainly centered around the polarizing effects that it can confer on gene expression (e.g., imprinting). However, the effects of DNA methylation on gene expression are far more nuanced and can be heavily context dependent.

Hypomethylation of the α-synuclein gene (*SNCA*) promoter region has been reported in the substantia nigra of PD patients in some studies (Jowaed et al., 2010; Matsumoto et al., 2010), yet it is not replicated in others (Richter et al., 2012; Guhathakurta et al., 2017). Epigenomic changes associated with other genes, including hypomethylation of *NPAS2* (Lin et al., 2012) and *CYP2E1* (Kaut et al., 2012) and hypermethylation of *PGC1-*α (Su et al., 2015) and the H1 haplotype of Tau (*MAPT*) (Coupland et al., 2014), have also been implicated in PD. More recently, epigenome-wide association studies (EWAS) have identified a concordant dysregulation of the methylome in the brain and blood of PD patients (Masliah et al., 2013). Two additional EWAS have identified sites of altered DNA methylation throughout the genome in blood samples of PD patients versus controls (Moore et al., 2014; Chuang et al., 2017), suggesting that PD is associated with altered methylation that can be detected in peripheral blood samples and raising the possibility that a peripheral epigenetic biomarker of PD could be possible. Wang et al. (2019) recently published a PD blood meta-analysis combining methylation and expression array datasets, though the data types were not from the same patients. We recently determined that specific epigenetic signatures also correlate with PD progression (Henderson-Smith et al., 2019), suggesting that peripheral DNA methylation may also have utility for the tracking of disease progression.

The primary functional consequence of DNA methylation is a resulting effect on the regulation of gene expression. Thus, altered DNA methylation would be predicted to alter mRNA expression levels as well. In the current work, we profiled the methylome of whole blood from PD and healthy age-matched controls using the Illumina Infinium 450K Human Methylation bead chip and also performed mRNA-seq on the same blood samples to identify DNA methylation loci that are associated with differential gene expression. We present two data types collected from a single well-defined patient cohort intended as preliminary information for a large-sample-size longitudinal PD biomarker study. Our findings provide information about coordinate epigenome-wide regulation of gene expression across PD.

## Methods

### Patient Demographics

All studies were approved by the institutional review boards in accordance with the Declaration of Helsinki. Informed consent was obtained from all participants. The study participants completed a comprehensive neurologic examination and have received a clinical diagnosis of PD (*n* = 15) or control (CON; *n* = 15) from board-certified specialists at the Mayo Clinic, Arizona. The PD and CON patients were 27 and 53% female, respectively. The average ages per group at sample collection were 68 ± 5.2 (CON) and 71.3 ± 7.1 (PD) years old. The PD individuals were at an earlier stage [Hoehn and Yahr = 1.89 ± 0.48; UPDRS Part 3 (off) = 13.9 ± 2.1] and were cognitively normal based on the Mini-mental Status Exam (MMSE) score of 29 or greater. The controls had no evidence of a movement disorder and had MMSE scores of 30 in all cases.

### Blood Collection and DNA and RNA Extraction

Peripheral blood was collected from patients using standard venipuncture techniques into PreAnalytiX PAXgene blood DNA and RNA tubes. The vacutainers were inverted several times and stored at −80°C. The samples were isolated from peripheral leukocytes, according to the manufacturer’s instructions, with the PAXgene DNA or RNA isolation kits (Qiagen). The isolated samples were stored at −20°C.

### Methylation Array Procedure

We examined methylation using Illumina Infinium HumanMethylation450K Beadchips as per the manufacturer’s protocol. Genomic DNA samples were first bisulfite-converted using the Zymo EZ DNA methylation kit, as per the manufacturer’s instructions, with the alternative incubation conditions specified in the protocol for compatibility with the Illumina Infinium Methylation Assays. The bisulfite-converted DNA was amplified, fragmented, precipitated, resuspended, and hybridized to bead chips following the manual’s protocol. Fluorescent staining was automated with Illumina’s Tecan system. The chips were coated and vacuum-dried for preservation before scanning to retrieve fluorescence intensity data, representing methylated or unmethylated positions, with Illumina’s iScan.

### Methylation Analysis

The ChAMP pipeline (Tian et al., 2017) for Illumina 450K methylation array was used for all the analyses following the default workflow. Raw data (.idat files) were loaded into the program, and multiple filtering steps were first applied to exclude probes with detection *p*-value (default > 0.01), probes with < 3 beads in at least 5% of samples per probe, all non-CpG probes contained in the dataset, all single-nucleotide polymorphism (SNP)-related probes, all multi-hit probes, and all probes located in sex chromosomes. Data were then normalized with BMIQ Method (Teschendorff et al., 2013), and batch effects were corrected by ComBat (Johnson et al., 2007). Cell proportion was calculated based on a reference DNA methylation profile, and cell type influence on the whole blood data was removed by RefbaseEWAS (Houseman et al., 2012). The “estimateCellCounts” function in minfi (Aryee et al., 2014) was used to estimate the proportional abundance of blood cell types in the each sample based on the intensity specific for inter-group comparisons as probes present in the array.

Differentially methylated promoters/regions (DMRs) were detected using the β values by the Limma (Ritchie et al., 2015) and BumpHunter method (Jaffe et al., 2012), respectively, as implemented in ChAMP. When technical replicates exist for the same sample, one data point was randomly picked for analysis.

### RNA Sequencing and Expression Analysis

mRNA was sequenced using the Illumina TruSeq RNA Library Prep kit on the HiSeq 2000 platform following the manufacturer’s protocol. Paired-end RNA reads were aligned to human genome reference (GRCh37 ensembl version 70), using STAR RNAseq read aligner (Dobin et al., 2013), and accepted mapped reads were summarized to gene level counts using the “featureCounts” function of the subread software package (Liao et al., 2013). Differentially expressed genes (DEGs) were reported by the DESeq2 package (Love et al., 2014) from the count matrix following standard protocol, using age + sex + RIN as covariates.

### eQTM Analysis

Expression quantitative trait methylation (eQTM) analysis was performed by applying the principle of expression QTL (eQTL) analysis to our DNA methylation and RNAseq datasets. Associations between differentially methylated loci and changes in gene expression were identified using the MatrixEQTL package (Shabalin, 2012) in R. eQTMs were measured in both *cis* (<1 Mbp from a gene) and *trans* (>1 Mbp from a gene).

### Functional Annotations

Gene ontology (GO) annotations and classifications were performed with the R package clusterProfiler (Yu et al., 2012) and web-based GeneMania (Montojo et al., 2010). Gene set enrichment analysis (GSEA) from the function “gseGO” was applied to the DEGs by setting nPerm = 1,000, minGSSize = 100, and maxGSSize = 500.

### Functional Network Analysis

Supervised functional network analysis was performed using an algorithm called functional epigenetic modules (FEM) in R, specifically to integrate the analysis of Illumina Infinium 450k array data with matched or unmatched gene expression data (Jiao et al., 2014). The statistics of differential DNA methylation and gene expression were obtained from “GenStatM” and “GenStatR” functions as implemented in FEM based on the normalized methylation and gene expression data matrices mapped to entrez ID. The normalized methylation data matrix was obtained from ChAMP. For the gene expression data matrix, genes with at least one count per million mapped reads in at least half of the sample libraries were retained first and then normalized using the “voom” function in the Limma package. Genes were mapped from ensembl ID to entrez ID using the R package biomaRt (Durinck et al., 2009). The protein interaction network used in the study includes only protein–protein interactions derived from Pathway Commons (Cerami et al., 2011) and was downloaded from http://sourceforge.net/projects/funepimod/files under filename “hprdAsigH^∗^.Rd.”

## Results

### Differential Expression

**FIGURE 1:**
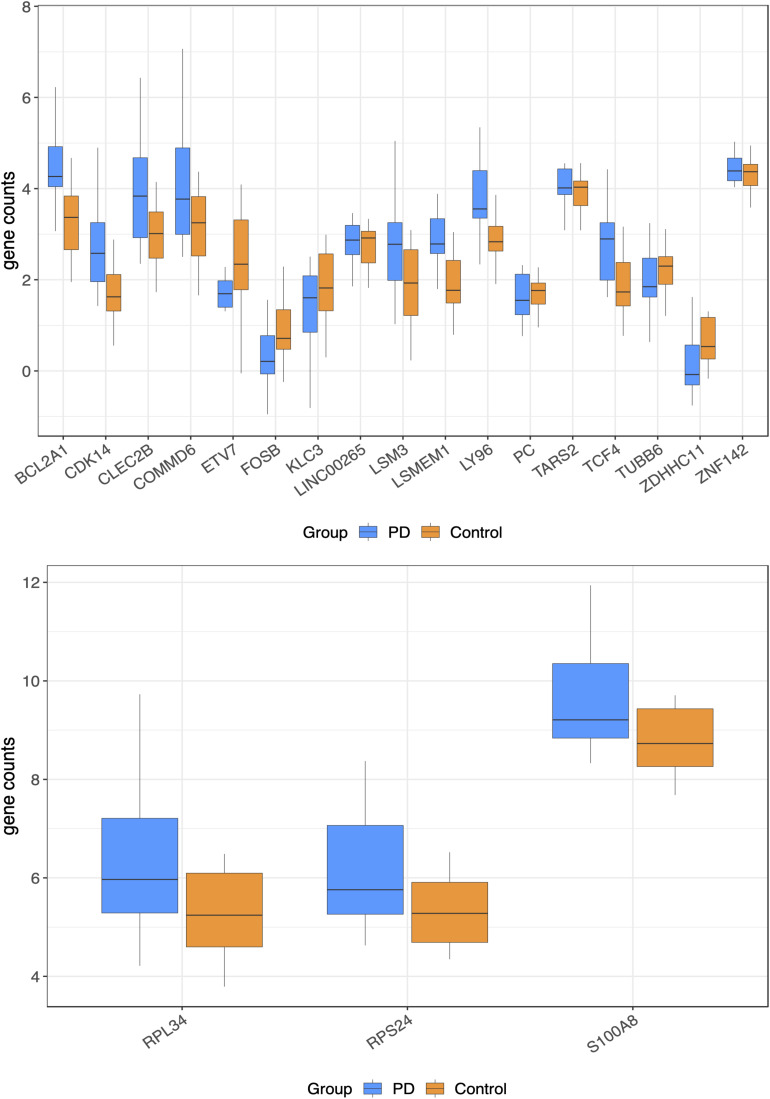
Normalized gene counts for the top 20 genes significant differentially expressed genes (the plots were divided for clearer viewing).

Differentially expressed genes in PD samples relative to controls are shown in Table 1 and Figure 1. Of the 526 DEGs, 30 were significant after multiple test correction (false discovery rate, FDR < 0.05). Gene set enrichment analysis showed three significant molecular functions for PD *vs*. control genes, which are related to nucleic acid binding (Table 2). Other top gene ontology terms for this group of genes were primarily related to cellular respiration and electron transport (Table 2). Data are available under GEO accession code GSE165083.

**TABLE 1:** Nine genes were under-expressed and 21 genes were overexpressed in Parkinson’s disease (false discovery rate ≤ 0.05).

**Gene**	**Log (fold change)**	***p***	***p* (adjusted)**
FOSB	−0.69	4.93E-05	0.048
ETV7	−0.68	4.95E-06	0.046
TUBB6	−0.60	7.32E-05	0.048
PC	−0.54	1.70E-05	0.048
ZDHHC11	−0.50	9.27E-05	0.048
KLC3	−0.48	9.55E-05	0.048
LINC00265	−0.43	7.21E-06	0.046
TARS2	−0.39	7.31E-05	0.048
ZNF142	−0.30	4.28E-05	0.048
RPS24	0.78	2.21E-05	0.048
CDK14	0.68	1.06E-04	0.048
CLEC2B	0.67	5.84E-05	0.048
RPL34	0.66	9.98E-05	0.048
BCL2A1	0.66	6.74E-05	0.048
TCF4	0.65	8.46E-05	0.048
COMMD6	0.64	5.25E-05	0.048
LY96	0.64	2.58E-05	0.048
LSM3	0.63	2.59E-05	0.048
S100A8	0.62	1.11E-04	0.048
LSMEM1	0.61	3.36E-05	0.048
COX6C	0.61	1.01E-04	0.048
COX7B	0.61	9.97E-05	0.048
RCAN1	0.61	2.59E-05	0.048
RNF175	0.59	6.96E-05	0.048
RAD51C	0.56	3.18E-05	0.048
FAM200B	0.49	4.75E-05	0.048
lnc-PDK3-1	0.46	8.66E-05	0.048
GLIPR1	0.45	4.59E-05	0.048
KIN	0.42	1.09E-04	0.048
NPEPPS	0.35	1.07E-04	0.048

**TABLE 2:** Gene set enrichment analysis (GSEA) molecular functions and gene ontology (GO) biological functions of differentially expressed genes.

**GSEA molecular functions**								**GO biological functions**			
**ID**	**Description**	**Set**	**Enrichment**	**Normalized**	***p***	**p**	***q***	**Function**	**False**	**Genes in**	**Genes in**
		**size**	**score**	**enrichment**		**(adjusted)**			**discovery**	**network**	**genome**
				**score**					**rate**		
GO:1901363	Heterocyclic compound binding	181	−0.22	−1.93	0.003	0.033	0.024	Respiratory electron transport chain	1.33E-11	11	103
GO:0097159	Organic cyclic compound binding	183	−0.21	−1.84	0.007	0.033	0.025	Electron transport chain	1.33E-11	11	104
GO:0003676	Nucleic acid binding	133	−0.21	−1.73	0.012	0.041	0.03	Cellular respiration	2.53E-10	11	140
								Mitochondrial inner membrane	1.49E-08	11	208
								Organelle inner membrane	1.93E-08	11	221
								Mitochondrial membrane	1.93E-08	12	294
								Hydrogen ion transmembrane transport	3.93E-08	7	44
								Energy derivation by oxidation of organic compounds	2.24E-07	11	285
								Proton transport	1.49E-06	7	75
								Hydrogen transport	1.62E-06	7	77
								Response to fungus	4.27E-03	3	11
								Monovalent inorganic cation transport	8.06E-03	7	273
								Lipopolysaccharide-mediated signaling pathway	8.59E-02	3	30

### Differentially Methylated Regions

**FIGURE 2:**
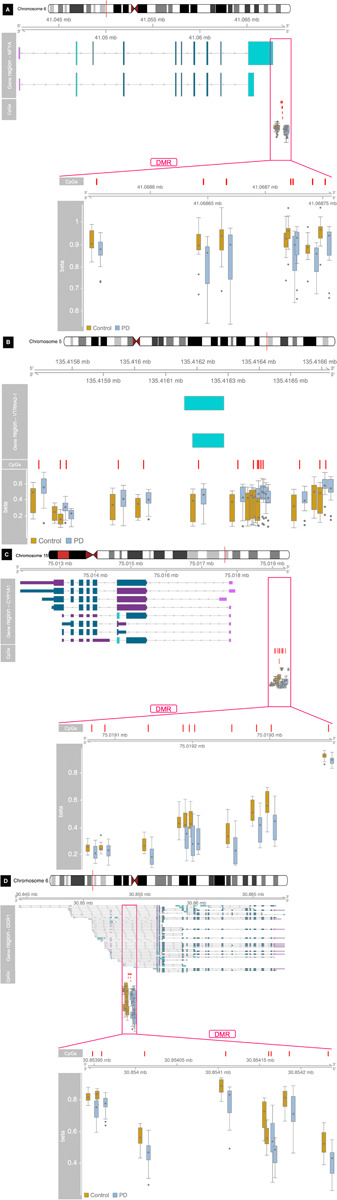
Differentially methylated regions nearest to the following genes: **(A)** *NFYA*, **(B)** *VTRNA2-1*, **(C)** *CYP1A1*, and **(D)** *DDR1*.

We found 31 differentially methylated regions. Thirteen regions were comprised of CpG sites that were hypermethylated in PD, and 18 regions were hypomethylated (Table 3). The DMRs were found in 13 chromosomes; chromosome 6 alone contained nine DMRs. DMR1 comprised seven CpG sites at *NFYA* (*p* = 3 × 10^–4^; Figure 2A). DMR5 contained 15 CpG sites that span the vault RNA2-1 (*VTRNA2-1*; *p* = 9 × 10^–4^) gene region (Figure 2B). Ten CpG sites for DMR15 were near cytochrome P450, family 1, subfamily A, polypeptide 1 (*CYP1A1*; *p* = 5.2 × 10^–3^; Figure 2C). DMR18 mapped to the discoidin domain receptor tyrosine kinase 1 (*DDR1*; *p* = 7.5 × 10^–3^) gene region where eight CpG sites were hypomethylated in PD (Figure 2D).

**TABLE 3:** Thirteen hypermethylated and 18 hypomethylated in Parkinson’s disease.

**Name**	**Region location**	**value**	**CpGs**	***p***	**Nearest genes**
DMR 2	chr6:32145146-32146779	0.40	37	0.0004	RNF5P1, RNF5, AGPAT1
DMR 4	chr2:239140032-239140340	1.43	7	0.0006	LOC151174, LOC643387
DMR 5	chr5:135415693-135416613	0.71	15	0.0009	VTRNA2-1
DMR 6	chr5:35617730-35618383	0.94	11	0.0013	SPEF2
DMR 7	chr1:75198211-75199117	0.91	11	0.0014	TYW3, CRYZ
DMR 11	chr6:31148332-31148666	0.53	14	0.0023	
DMR 17	chr7:130419042-130419514	0.40	14	0.0057	KLF14
DMR 16	chr1:205818956-205819609	0.80	7	0.0065	PM20D1
DMR 22	chr2:54086854-54087343	0.53	11	0.0068	GPR75, LOC100302652
DMR 19	chr10:49812686-49813208	0.77	7	0.0074	ARHGAP22
DMR 20	chr2:183943181-183943551	0.69	8	0.0078	DUSP19
DMR 25	chr22:45809523-45810043	0.44	10	0.0151	SMC1B, RIBC2
DMR 28	chr5:158689930-158690540	0.60	7	0.0158	UBLCP 1
DMR 1	chr6:41068553-41068752	−1.59	7	0.0003	NFYA
DMR 3	chr6:30038754-30039408	−0.40	27	0.0005	RNF39
DMR 8	chr6:31691597-31692375	−0.47	18	0.0018	C6orf25
DMR 10	chr1:223566127-223567173	−0.76	11	0.0025	C1orf65
DMR 9	chr6:74104097-74104868	−0.98	8	0.0025	DDX43
DMR 12	chr6:31275551-31275881	−0.74	10	0.0030	
DMR 13	chr7:27169674-27170994	−0.41	18	0.0031	HOXA4
DMR 14	chr6:33084420-33084933	−0.41	17	0.0031	HLA-DPB2
DMR 15	chr15:75019070-75019376	−0.63	10	0.0052	CYP1A1
DMR 18	chr6:30853948-30854233	−0.69	8	0.0075	DDR1
DMR 21	chr17:7832479-7833237	−0.68	8	0.0081	KCNAB3
DMR 23	chr3:10149803-10150139	−0.57	9	0.0105	C3orf24
DMR 24	chr18:23713595-23714084	−0.52	9	0.0140	PSMA8
DMR 31	chr10:77542354-77542585	−0.59	7	0.0168	C10orf11
DMR 27	chr8:144659831-144661051	−0.48	9	0.0178	NAPRT1
DMR 26	chr11:67383377-67384040	−0.53	8	0.0184	
DMR 29	chr10:4868328-4868690	−0.51	8	0.0205	AKR1E2
DMR 30	chr1:1108820-1109832	−0.50	8	0.0227	TTLL10

### eQTM for Parkinson’s Disease

**FIGURE 3:**
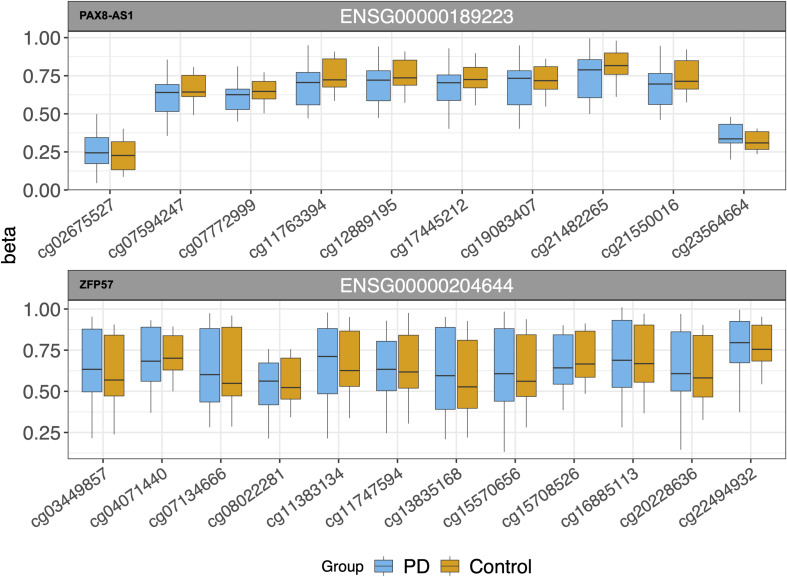
*cis*-eQTMs for multiple CpG sites near two genes (*PAX8-AS1* and *ZFP57*).

Our eQTM analysis showed 27 *cis* and 67 *trans* CpG sites associated with differential gene expression. After FDR correction (FDR ≤ 0.05), 19 *cis* and 43 *trans* eQTMs remained significant. In the *cis*-eQTM set, there were two genes associated with the majority of CpG sites: PAX8 antisense RNA1 (*PAX8-AS1*) and zinc finger protein 57 (*ZFP57*) (10 and 12 sites, respectively, Table 4 and Figure 3). Solute Carrier Family 29 Member 1 (*SLC29A1*, a.k.a. *ENT1*) was an additional gene of interest in the *cis*-eQTMs for its role in Alzheimer’s and Huntington diseases (Guitart et al., 2016; Lee et al., 2018), though it did not pass significance after multiple test correction (FDR = 0.07; Table 4). There were five genes in the *trans* set that made up the majority of eQTMs (Table 5): C-type lectin domain containing 11A (*CLEC11A*), signal-induced proliferation-associated 1, like 2 (*SIPA1L2*), ribosomal protein L29 (*RPL29* pseudogene/AL450405.1), and T cell receptor beta variable 11-2 (*TRBV11-2*). Multiple genes are generally expected to be associated with some of the same single CpG sites. Therefore, an individual gene associated with multiple CpG sites may be suggestive of a gene under tighter regulatory control.

**TABLE 4:** *cis*-eQTM sites (< 1 Mbp from a gene) with false discovery rate (FDR) < 0.05 are shown, with the exception of three sites corresponding to *ZFP57* and an additional gene of interest (*ENT1*).

**CpG**	**Gene**	**Statistic**	***p***	**FDR**
cg19083407	PAX8-AS1	−11.52	1.53E-10	0.001
cg23564664		10.78	5.13E-10	0.002
cg07594247		−10.43	9.29E-10	0.002
cg17445212		−10.33	1.10E-09	0.002
cg12889195		−10.00	1.93E-09	0.003
cg07772999		−9.22	7.87E-09	0.01
cg11763394		−9.13	9.34E-09	0.01
cg21550016		−9.09	9.97E-09	0.01
cg02675527		9.04	1.10E-08	0.01
cg21482265		−8.81	1.69E-08	0.01
cg15708526	ZFP57	−9.69	3.34E-09	0.005
cg07134666		−9.46	5.11E-09	0.01
cg15570656		−9.35	6.24E-09	0.01
cg16885113		−8.95	1.29E-08	0.01
cg08022281		−8.81	1.69E-08	0.01
cg04071440		−8.44	3.43E-08	0.02
cg11383134		−8.23	5.22E-08	0.02
cg03449857		−8.19	5.68E-08	0.03
cg20228636		−8.03	7.76E-08	0.03
cg13835168		−7.70	1.51E-07	0.06
cg22494932		−7.68	1.57E-07	0.06
cg11747594		−7.44	2.60E-07	0.08
cg23228858	ENT1	−7.53	2.15E-07	0.07

**TABLE 5:** *trans*-eQTM sites (>1 Mbp from a gene), false discovery rate (FDR) ≤ 0.05.

**CpG**	**Gene**	**Statistic**	***p***	**FDR**
cg13291296	CLEC11A	−12.09	6.34E-11	0.02
cg18894521		−11.04	3.31E-10	0.05
cg21028260		−10.98	3.70E-10	0.05

cg11460641	SIPA1L2	−13.48	8.30E-12	0.01
cg26287080		−12.69	2.58E-11	0.01
cg21622464		−12.23	5.13E-11	0.02
cg24297509		−11.57	1.42E-10	0.03
cg26914392		−11.44	1.76E-10	0.03

cg11199182	TRBV19	−11.92	8.30E-11	0.02
cg25335258		−11.67	1.22E-10	0.03
cg11342941		−11.35	2.03E-10	0.03
cg04446870		−10.97	3.72E-10	0.05
cg17153055		11.32	2.12E-10	0.03

cg26835568	AL450405.1/(RPL29) pseudogene	−13.92	4.52E-12	0.01
cg01772663		−12.82	2.13E-11	0.01
cg02810976		−11.77	1.04E-10	0.03
cg21475003		−11.70	1.16E-10	0.03
cg08272591		−11.68	1.19E-10	0.03
cg06312137		12.16	5.70E-11	0.02
cg00266592		11.54	1.49E-10	0.03
cg25593954		11.54	1.49E-10	0.03
cg12067423		11.52	1.53E-10	0.03
cg02942845		11.24	2.40E-10	0.03

cg16515546	TRBV11-2	−16.66	1.40E-13	0.00
cg13291296		−13.56	7.40E-12	0.01
cg21950182		−12.39	4.00E-11	0.02
cg17731973		−12.06	6.65E-11	0.02
cg15614872		−11.98	7.48E-11	0.02
cg06809285		−11.62	1.31E-10	0.03
cg21028260		−11.35	2.02E-10	0.03
cg18696632		14.13	3.40E-12	0.01
cg18803655		13.85	4.96E-12	0.01
cg21475544		13.63	6.74E-12	0.01
cg21300072		12.73	2.44E-11	0.01
cg01840459		11.28	2.26E-10	0.03

cg08029281	RPL26L1	11.29	2.22E-10	0.03
cg22128645	GEMIN8	11.57	1.41E-10	0.03
cg08029281	HSPB11	12.27	4.83E-11	0.02
cg25751895	PCSK1N	11.48	1.64E-10	0.03
cg24512544	ACOT11	−12.21	5.29E-11	0.02
cg08029281	TOMM7	10.98	3.71E-10	0.05
cg22793142	TTN-AS1	−11.37	1.94E-10	0.03
cg12609785	DND1P1	11.37	1.95E-10	0.03

### Functional Epigenetic Module Modeling

**FIGURE 4:**
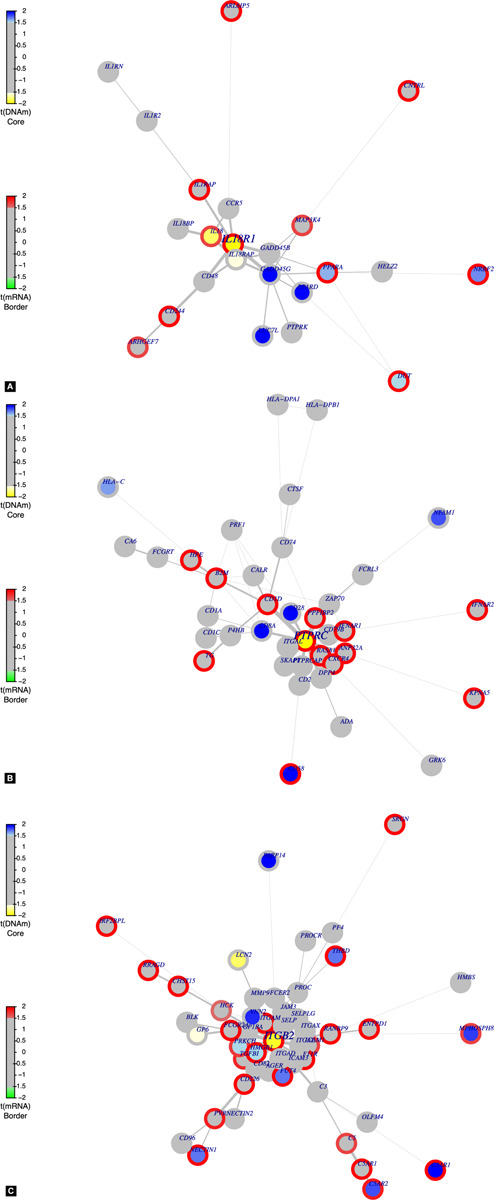
Functional epigenetic protein–protein interaction modules for **(A)** IL18R1, **(B)** PTPRC, and **(C)** ITGB2.

FEM network analysis suggested likely gene targets of nearby methylated sites by identifying inversely correlated expression and methylation levels, verified against regions in a PPI network that reflect similar patterns. There were six protein modules as follows: interleukin 18 receptor 1 (IL18R1, *p* = 0.001; Figure 4A), protein tyrosine phosphatase receptor type C (PTPRC, *p* = 0.031; Figure 4B), MOB family member 4, phocein (MOB4, *p* = 0.036), integrin subunit beta 2 (ITGB2, *p* = 0.021; Figure 4C), MYB proto-oncogene, transcription factor (MYB, *p* = 0.037), and SMAD-specific E3 ubiquitin protein ligase 2 (SMURF2, *p* = 0.018; Table 6).

**TABLE 6:** Functional epigenetic modules from protein–protein interaction networks.

**Gene (seed)**	**Size (number of genes)**	**Mod**	***p***	**Sampling of genes in network**
IL18R1	23	1.83	0.001	IL18R1, GADD45B, IL1RAP, GADD45G, IL18RAP, CCR5, IL18, CD48, IL18BP, PTPRK
SMURF2	45	1.86	0.018	SMURF2, TNNT1, NEK6, TRAF4, TNFRSF1B, RNF111, RAP1B, TCAP, CCAR1, IRF8
ITGB2	50	1.42	0.021	ITGB2, TGFBI, C3, ICAM1, ITGAM, PRKCH, MMP9, GP1BA, CD82, HCK
PTPRC	38	1.25	0.031	PTPRC, ZAP70, CD1D, CD79B, ANP32A, RASA1, IFNAR1, CXCR4, CD8A, CD2
MOB4	11	1.78	0.036	MOB4, STK11, SCMH1, KIDINS220, EGLN2, PTPRS, UBE2V2, SNRK, LYPLA2, STK11IP
MYB	22	1.83	0.037	MYB, CDK6, PIM1, PPP3CA, MAF, PTGS2, PPID, ATP2B1, PTCRA, TOM1

*The direction of expression for the seed genes is inverse to the methylation levels nearest to them.*

## Discussion

In this study, we collected whole blood samples from a small cohort of PD patients and controls, carried out RNAseq and DNA methylation assays, and performed comprehensive and complete analysis of the data. In the DNA methylation data analysis, a rigorous quality control workflow was applied, with batch effects from methylation array position explicitly removed by ComBat (Johnson et al., 2007) and cell proportion adjusted for by using a reference database of DNAm profiles of the major cell types presented in whole blood, as implemented in RefbaseEWAS (Houseman et al., 2012). An analysis by singular value decomposition of the final dataset shows no strong correlation between deconvoluted components and covariates (Supplementary Figure 1); thus, in the downstream analysis, no covariates were included.

Important pathways and genes currently known in the search for PD etiology were identified in this study, even with the relatively small sample size. RNA expression and DNA methylation were queried from blood samples of age- and gender-matched PD and control groups. Analysis of the resulting data and identification of previously known genes contribute a critical step in reinforcing suspected mechanisms associated with the disease. In addition, genes and regulatory sites previously unassociated with PD, or any other neurodegenerative disorder, were identified. In the following sections, we present brief highlights of the genes and sites of interest as revealed in our results that exhibit important functional characteristics and potential disease relevance, keeping in mind that these peripheral tissue (whole blood) results may not be fully reflective of the disease etiology in the brain. However, our findings provide clues for future work and larger follow-up studies aimed at achieving a blood-based biomarker to predict, diagnose, evaluate, and possibly track the progression of PD.

### Differentially Expressed Genes

We identified ∼30 DEGs in the PD group following multiple test correction. Many of these genes are transcription factors, e.g., *FOSB*, *ETV7*, *TCF4*, *ZNF142*, etc., suggesting dysregulated peripheral gene expression in PD. Among these was fosB proto-oncogene, AP-1 transcription factor subunit (*FOSB*), an immediate early gene and also a regulator of Parkin expression in brain tissues (Patterson et al., 2016). In dopaminergic pathways, *FOSB* functions along the mesocorticolimbic projection in the reward circuitry and along the nigrostriatal pathway affecting motor function (Manning et al., 2017). Striatum expression levels are affected in L-DOPA-induced dyskinesia (LID) during L-DOPA treatment. In LID, reduced G-protein coupled receptor kinase (GRK) expression leads to the supersensitivity of dopamine (DA) receptors, activation of the ERK pathway, and accumulation of Δ*FOSB* (truncated isoform). Correction of DA receptor sensitivity with GRK administration reduced Δ*FOSB* accumulation along with the severity of LID in mice (Ahmed et al., 2019). These findings suggest that *FOSB* plays a role in PD pathogenesis. A few other DEGs are also implicated in the neurodegenerative process. Pyruvate carboxylase (PC) is an important astrocyte-specific enzyme linked to AMPK within the TCA cycle in the brain (Voss et al., 2020). It plays a vital role in the nervous system, where it replenishes the building blocks needed to make neurotransmitters. Additionally, PC is necessary for the formation of myelin to insulate and protect certain nerve cells (Garcia-Cazorla et al., 2006).

Disruption of mitochondrial homeostasis has been demonstrated as a crucial cofactor in PD etiology (Dolle et al., 2016). Functional enrichment analysis of the DEGs highlights the important pathways that are dysregulated in the blood of PD patients, which includes two cytochrome C oxidase (*COX*) genes that are related directly to altered mitochondrial function (Table 2). *COX7B* has previously been implicated in PD as a differentially expressed gene, though it was downregulated, contrary to our result for this gene (Kong et al., 2018). Our work calls for more attention to the understanding of neurobiology and signaling networks operating on the mitochondria to identify the mechanisms responsible for oxidative damage and, subsequently, the loss of dopaminergic neurons (Scorziello et al., 2020).

### Differentially Methylated Regions

A total of 31 significant DMRs were identified in the PD patients’ methylation profiles, with 18 being hypomethylated. Differential DNA methylation does not necessarily directly result in the differential expression of the nearest genes in the genome. Nevertheless, it still provides insights into the regulatory machinery and disease processes that have potential use as biomarkers (Levenson, 2010). In the hypomethylated regions, some top CpG sites were located at the promoter region of *NFYA*. As one of the subunits composing the trimeric complex NF-Y, a highly conserved transcription factor that binds to CCAAT motifs in the promoter regions in a variety of genes, NFYA has been suggested as the regulatory subunit to confer sequence specificity for the binding of the complex to the DNA transcriptional start site (TSS) (Oldfield et al., 2014). NFYA is reduced in some polyglutamine expansion neurodegenerative diseases, and conditional deletion in mouse neurons induces neurodegeneration with ubiquitin/p62 accumulation (Yamanaka et al., 2014). Discoidin domain receptor (*DDR1*) was also hypomethylated in our PD group. *DDR1* codes for a receptor tyrosine kinase containing a conserved collagen-binding domain. Nilotinib tyrosine kinase inhibitor works potently against DDR1 and is used to treat adults with chronic myeloid leukemia. A low-dose administration of this inhibitor degraded misfolded alpha-synuclein and increased dopamine levels in animal models of neurodegenerative diseases (Pagan et al., 2019). Pagan et al. (2019) also found that nilotinib increases the cerebrospinal fluid (CSF) levels of TREM2 and potentially reduces oligomeric alpha-synuclein in human PD patients. Mo et al. (2019) looked for causal associations of sites and genes in multiple sclerosis (MS) and found multiple SNPs, DNA methylation sites, and differential gene expression for *DDR1*. They also identified an MS-associated methylation site near *AGPAT1*, another DMR identified in the present work (Mo et al., 2019).

The top hypermethylated CpG sites were located near the promoter regions of multiple genes. Both *RNF5* and 1-acylglycerol-3-phosphate O-acyltransferase 1 (*AGPAT1*) have been implicated in neurodegenerative diseases. Emerging evidence suggests that, besides being an age-associated disorder, PD might also have a neurodevelopmental component. Agarwal e*t al*. found subtle, though non-significant, abnormalities in the hippocampal development of their *AGPAT1* knockout mice, which they investigated in light of knockout-induced audiogenic seizures (known to originate from the CA region of the hippocampus). Additionally, these mice exhibited hypoglycemia and reproductive abnormalities (Agarwal et al., 2017). SNPs at the *AGPAT1* locus have recently gained attention in several areas of disease study. One SNP near *AGPAT1* demonstrated a negative allelic effect between frontotemporal dementia, which exhibits parkinsonism and related pathology, and (immune-mediated) celiac disease; another SNP has been associated with Alzheimer’s disease (Agarwal et al., 2017; Broce et al., 2018; Rowe, 2019). RNF5 is an important player in muscle physiology and ER stress dysregulation, and it is found in cytoplasmic aggregates of an acquired myopathy common in older people, including body myositis (IBM), along with tau and amyloid-β. This pathology paired with similarities between IBM muscle fibers, and brains of Parkinson’s and Alzheimer’s disease patients present an interesting link to neurodegeneration (Delaunay et al., 2008; Askanas and Engel, 2011).

There was hypermethylation in PD of 15 CpG sites spanning the vault RNA2-1 (*VTRNA2-1*) gene. In 2013, a small non-coding RNA, considered to be a fragment of *VTRNA2-1*, was characterized and named small VTRNA2-1 (sVTRNA2-1). sVTRNA2-1 was upregulated (1.5-fold) in the amygdala (AM), SN (2.5-fold), and frontal cortex (twofold) of late-stage (4 and 5) PD patients, independent of the expression levels of full-length VTRNA2-1. Upregulation (twofold) was also found in pre-clinical AM cases (Minones-Moyano et al., 2013). Cytochrome P450, family 1, subfamily A, polypeptide 1 (*CYP1A1*), involved in xenobiotic metabolism, is a target for aryl hydrocarbon receptor, which may influence Parkin expression (Gonzalez-Barbosa et al., 2019). The *CYP1A1 M1* polymorphism is associated with increased PD risk in men (Kumudini et al., 2014).

### Expression Quantitative Trait Methylation

We tested associations between CpG probes within 1 Mb of the TSS of differentially expressed genes (measured by RNA-seq) in order to identify sites with correlation between DNA methylation and expression from a nearby gene (*cis*-eQTM). This is the first time PAX8 antisense RNA1 (*PAX8-AS1*), a long non-coding RNA, has been reported to be associated with a neurodegenerative disorder. Most recently, it was identified as an apoptosis regulator in diabetic neuropathy (Shen et al., 2020). Zinc finger protein 57 (*ZFP57*) has not been previously linked to a neurodegenerative disorder, but its involvement in neurological health is evidenced by research in post-traumatic stress disorder (PTSD). Two recent studies link *ZFP57* DNA methylation levels to PTSD symptoms, with the first identifying a longitudinal decrease in methylation at this gene (Rutten et al., 2018). The second study showed that PTSD treatment lead to increased regional methylation in *ZFP57* which was related to symptom reduction (Vinkers et al., 2019). The relationship between DNA methylation, gene expression, and pathogenesis in these findings certainly awaits in-depth study.

### Functional Epigenetic Module Analysis

By integrating DNA methylation data with gene expression in a systems context using a human PPI network as a scaffold, we were able to identify candidate gene modules whose differential expression is regulated by differential methylation in PD. As hotspots in the interactome, several proteins whose expression was inversely correlated with methylation values were identified, which may show the most direct functional relationships across the two datasets. The most prominent PPI hotspots identified in our analysis are the IL18/IL18R1 pathway (*p* < 0.01, Figure 4A), which clearly shows an upregulation of IL18/IL18R1 expression through hypomethylation. Recent studies have pinpointed a critical role for IL-18 in mediating neuroinflammation and neurodegeneration in the central nervous system under pathological conditions (Felderhoff-Mueser et al., 2005; Alboni et al., 2010). In the 1-methyl-4-phenyl-1,2,3,6-tetrahydropyridine model of PD, the duration of microglial response was shorter in IL18^–/–^ mice, emphasizing the contribution of IL18 to neuroinflammatory processes in PD (Machado et al., 2016). Two other genes were also previously found together as part of an immune/microglial module in a weighted gene co-expression network analysis, protein tyrosine phosphatase receptor type C (*PTPRC*) and integrin subunit beta 2 (*ITGB2*) (Mukherjee et al., 2019). In separate studies, PTPRC expression was downregulated in living PD patients, one in CSF and one in blood (Hossein-Nezhad et al., 2016). The interactome hotspots located by integrating transcriptome and methylome profiles demonstrated that immune responses for the pathology could be detected in the patients’ blood, therefore opening a channel for studying disease mechanism and discovering blood-based biomarkers in the future.

## Conclusion

We presented analyses of differential gene expression and differential methylation data, leveraging the potential of gathering and combining two data types that represent highly interconnected biological processes. The individual datasets yielded some interesting results alone. However, the combined analyses (eQTM and FEM) identified events that may be more functionally relevant since the genes discussed were not only differentially expressed but also more likely to be under the influence of disease-specific DNA methylation changes. This study is a critical intermediate step toward defining the most impactful sites to be considered for downstream biomarker development.

## Data Availability Statement

The datasets presented in this study can be found in online repositories. The names of the repository/repositories and accession number(s) can be found below: NCBI Gene Expression Omnibus, accession no: GSE165083.

## Ethics Statement

The studies involving human participants were reviewed and approved by Institutional Review Board. The patients/participants provided their written informed consent to participate in this study.

## Author Contributions

AH contributed to laboratory work, interpretation of data, data analysis, and manuscript drafting and revision. QW contributed to data analysis, interpretation of data, and manuscript drafting and revision. BM and AS contributed to laboratory work and experimental design. MD and MN contributed to data analysis. MH contributed to manuscript revision for intellectual content. RC and ED-D contributed to study design and sample procurement. TD contributed to study conceptualization and design, interpretation of data, and manuscript drafting and revision.

## Conflict of Interest

The authors declare that the research was conducted in the absence of any commercial or financial relationships that could be construed as a potential conflict of interest.

## Supplementary Material

Supplementary Figure 1 Singular value decomposition analysis plot after batch correction, showing the association of covariates with the most significant components of variation for the beta matrix.
