# Supplementary figures and images for: DNA Methylation and Expression Profiles of Whole Blood in Parkinson’s Disease
Source: Front Genet. 2021 Apr 26;12:640266. doi: 10.3389/fgene.2021.640266 (PMC8107387; doi:10.3389/fgene.2021.640266)

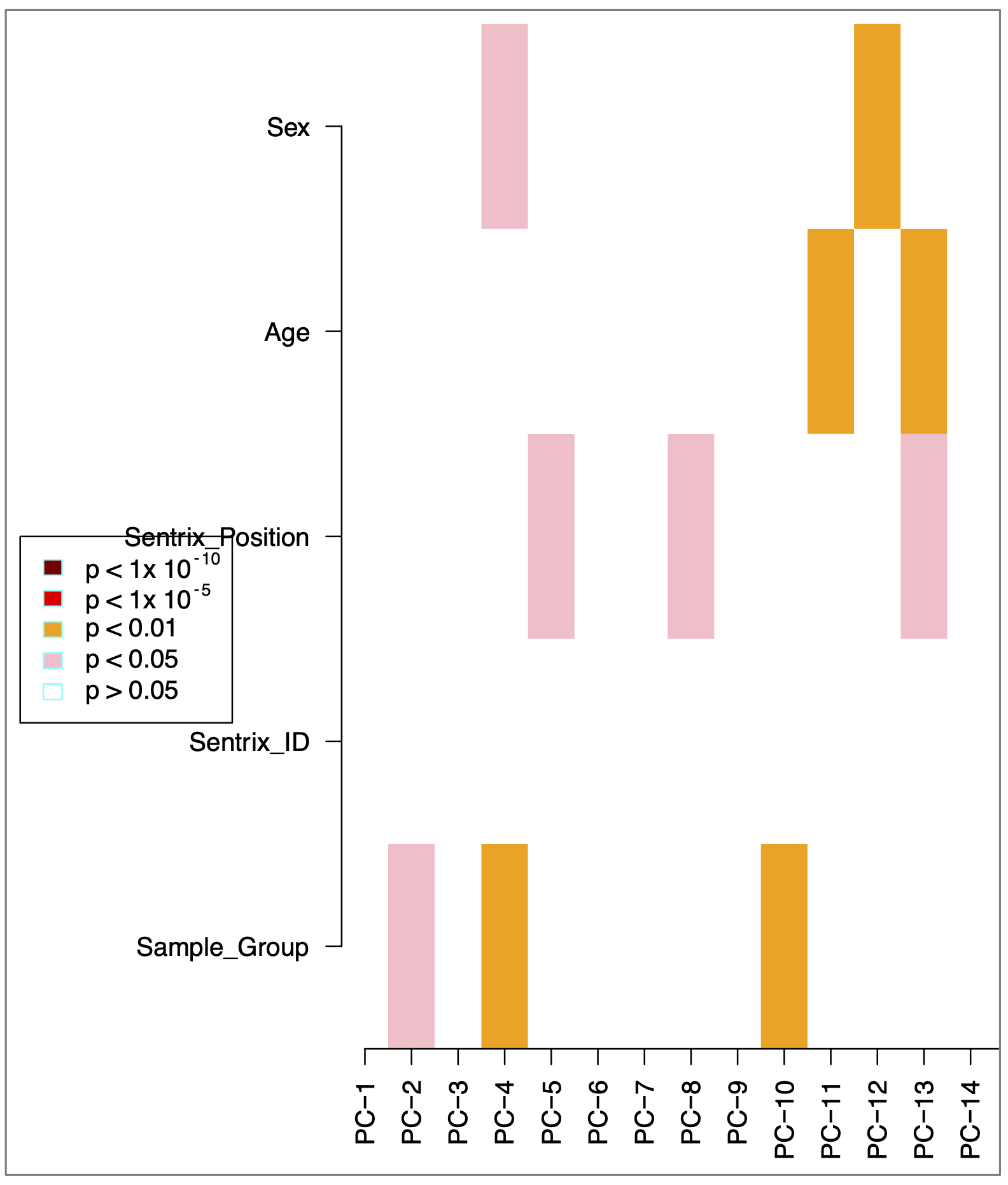

Supplement: Supplementary Figure 1 — Singular value decomposition analysis plot after batch correction, showing the association of covariates with the most significant components of variation for the beta matrix. [file Image_1.TIFF]
